# Perceived risk of reinfection among individuals treated for sexually transmitted infections in Northern Ethiopia: implication for use in clinical practice

**DOI:** 10.11604/pamj.2017.27.87.12015

**Published:** 2017-06-05

**Authors:** Mache Tsadik, Yemane Berhane, Alemayehu Worku, Wondwossen Terefe

**Affiliations:** 1 College of Health Science, Mekelle University, Tigray, Ethiopia; 2 Addis Continental Institute of Public Health, Addis Ababa, Ethiopia; 3 Addis Ababa University, Addis Ababa, Ethiopia

**Keywords:** Ordinal regression, risk perception, STI reinfection

## Abstract

**Introduction:**

The prevention of reinfection of sexually transmitted infections (STIs) is highly dependent on the level of risk perception and the subsequent adoption of preventive behaviors. While perceived risk is assumed to be key to adoption of preventive measures, the evidence regarding the predictors of perceived risk to STI reinfection are limited.

**Methods:**

This paper is based on a cross sectional facility based survey conducted in North Ethiopia from January to June; 2015. Patients attending public health facilities for STI care responded to a structured questionnaire at clinic exist. Ordinal logistic regression was employed to identify factors associated with risk perception.

**Results:**

Of the 1082 STI patients who participated in the study, 843(77.91%) indicated a high perceived risk of STI reinfection. The major factor associated with low perceived risk of reinfection was willingness to notify partner; the odds of being willing to notify partner was greater among those who perceived low risk (AOR=3.01, 95% CI: 2.13-4.25). In addition, low perceived risk was associated with female index cases (AOR=1.49, 95% CI: 1.07-2.08), those who had high school education and above (AOR=1.68, 95% CI: 1.07-2.65), those aged 25 years and above (AOR=1.52, 95% CI: 1.09-2.12), those who had a single partner (AOR=1.82, 95% CI: 1.20-2.74), and those who had low perceived stigma (AOR=1.42, 95% CI: 1.04-1.95).

**Conclusion:**

The perceived risk of STI reinfection is high and strongly associated with willing to notify partner. Efforts to prevent STI reinfection need to consider interventions that enhance partner notification.

## Introduction

Sexually transmitted reinfection refers to a second occurrence of the same infection, typically from untreated infected partner [1]. Partner notification (PN) has a major role in preventing reinfection and decrease the pool of infectious people in the community [2, 3]. The reinfection of sexually transmitted infections (STIs) reflects the inadequacy of partner management in health system. Reinfection suggests the existence of risk behaviors that have not been modified after treatment [4]. The proportion of STIs reinfection ranges from 6%-30%, it also shows considerable variation by type of infection [5–8]. Reinfection after treatment for *curable STIs is becoming a serious problem as it* reduces the effect of control interventions [9]. This indicates providing improved prevention and curative services for patients with STIs and their sexual partners who are at elevated risk of reinfection is paramount. Perceived risk is crucial for the assessment of actual sexual behaviors though it is a complex multi factorial process influenced by socioeconomic, political and cultural contexts [10]. The way individuals perceive and respond to risk depends on the individual level of awareness and control of other influencing factors [11] and the extent to which it affects individuals health seeking behavior [12, 13]. For instance, a facility based study revealed that more than a third of patients with prior STIs, do not perceive themselves as at risk for another STI, and choose not to use condoms [14]. Perceived risk of reinfection for STIs is an important indicator for the likelihood of preventive action to be taken by the patient. However, information regarding the predictors of perceived risk to STIs reinfection is limited in Africa. Thus, this study was conducted to identify factors associated with STIs reinfection risk perception among patients visiting health facility for STI care in northern Ethiopia.

## Methods

### Study setting

This study was conducted in public health facilities in Tigray region. The syndromic management was the standard mode of STI clinical care in Ethiopia at all levels. STI related services are provided in an integrated manner with the routine care in all service delivery points in Ethiopia.

### Study design

This was a facility based cross-sectional study conducted from January to June 2015. Based on the Health Management Information System (HMIS) report of Regional Health Bureau in 2013/2014, health facilities with a minimum monthly load of five syndromically diagnosed STI cases were included in the study. All STIs cases attending the selected health facilities for STI care during the study period were included in the study.

### Data collection

The data collection tool for this study was developed based on standard questionnaires used previously [15]. The questionnaire was pretested in the context of this study. Data collectors were nurses drawn from health facilities. Three days training was given to data collectors regarding the objective of the study, patient’s confidentiality and privacy, interview technique, patient’s right to refuse and appropriate recording and data handling. Eligible patients were linked to the study team by the treating health workers at clinic exist. All interviews took place in a separate room after getting informed consent.

### Measurement

Measurement of risk perception to reinfection was adopted from previous study [16]. A single item question was asked - “How large is your risk of getting STI again from your untreated partner/s?” The question had five response categories- no risk (1), small risk (2), some risk (3), high risk (4), and very high risk (5). The outcome variable was orderly categorized in to three responses as “low risk” (coded as “0”) by merging the responses of “no risk and small risk”, “medium risk” coded as “1” for the response of some risk, and “high risk” coded as “2” for those responded “high risk and very high risk”. The independent variables assessed in this study included socio-demographic, behavioral and psychosocial factors. The sociodemographic variables were Age categorized as < 24, > 25; Sex coded as male and female; Marital status coded as married and single; Educational status coded as illiterate, primary, and secondary+; current residence coded as urban and rural. Sexual behavioral variables were partnership status coded as regular and casual; Number of partners within three months coded as single and multiple; Condom use within three months coded as yes and no; and New partner within three months coded as yes and no. Perceived stigma was measured using four item questions: 1) Referring a partner for STI treatment is shameful; 2) Attending health facility for STI treatment is embarrassing; 3) A good man/women go to health facility for STI treatment 4) A good man/women notify his/her partner. The response for each item had 5 scales in a range of strongly disagree (1), to strongly agree (5). It was classified in to high for those who scored above the mean and “low” for those who scored below mean (Cronbach’s alpha= 0.64). A single question was asked to index cases “How likely are you to notify/refer your sexual partner to the health facility within the next one week?” The response ranges from very unlikely (1) to very likely (4). It was dichotomized in to “unwilling to notify” coded as “0” by merging those who respond very unlikely and unlikely and “willing to notify” coded as “1” by merging those who respond very likely and likely. Self efficacy to prevention of reinfection was measured using three item questions: 1) How confident you feel to refuse your partner sex if your partner refuses to use condoms? 2) How confident you feel to convince your partner(s) to use condoms during sex? 3) How confident you feel about convincing each partner to get an STI check-up? The response for each item had 4 scales in a range of very unlikely (1), to very likely (4). This was classified in to high for those who scored above the mean and “low” for those who scored below mean (Cronbach’s alpha=0.94).

### Data analysis

Statistical analysis was done using the statistical package STATA version 12. Initially, we checked the association of independent variables with the outcome variable using Pearson’s chi-square at p-value < 0.05 to select potential variables as indicated in Table 1. Then, we run proportional odds model using the ologit command. The proportional odds models are suited for the analysis of ordinal response variables; however, it often fails to satisfy the critical parallel line assumption. This assumption was assessed in preliminary analyses, using Brant test. The test showed violation of the parallel slopes assumption (X2 = 35.47, p-value < 0.000) on three covariates as shown in Table 2. Thus, further analysis was done using a multivariate ordered logistic regression fitted in the context of the partial proportional odds model (PPOM). The AUTOFIT option with GOLOGIT2 is used to fit partial proportional odds models, where the parallel-lines constraint is relaxed only for those that met the assumption. Global Wald test for the final model indicates that the final model does not violate the proportional odds assumption with high p-value: 0.2211 as presented in Table 3.

**Table 1:** Patients’ perceived risk of STI reinfection by selected independent variables, North Ethiopia, 2015

Variables	Response	Risk perception to reinfection			Pearson chi-square (p value)
		Low risk N (%)	Medium risk N (%)	High risk N (%)	
***Sociodemographic factors***					
Age in completed years	≤ 24	87(15.99)	52 (9.56)	405(74.45)	7.93(0.019)^+^
	≥25	59(10.97)	41(7.62)	438(81.41)	
Sex of participant	Male	78(16.15)	47(9.73)	358(74.12)	7.48(0.024)^+^
	Female	68(11.35)	46(7.68)	485(80.97)	
Marital status	Married	53(10.27)	40(7.75)	423(81.98)	10.00( 0.005)^+^
	Single	93(16.43)	53(9.36)	420(74.20)	
Educational status	Illiterate	29(15.26)	26(13.68)	135(71.05)	17.83(0.001)+
	Primary	57(17.59)	21(6.48)	246(75.93)	
	High school+	60(10.57)	46(8.10)	462(81.34)	
Current residence	Urban	110(13.66)	53(6.58)	642(79.75)	16.24(0.001)^+^
	Rural	36(13.00)	40(14.44)	201(72.56)	
***Behavior related factors***					
Partnership type	Regular	66(10.38)	44(6.92)	526(82.70)	20.70(0.001)^+^
	Casual	80(17.94)	49(10.99)	317(71.08)	
Number of partners	Multiple	31(13.65)	20(8.21)	93(78.14)	17.15(0.001)^+^
	One	115(12.50)	73(11.11)	750(76.39)	
Condom use within 3month	Yes	32(14.10)	17(7.49)	178(78.41)	0.49(0.780)
	No	114(13.33)	76(8.89)	665(77.78)	
New partner within 3mon	Yes	34(16.50)	19(9.22)	153(74.27)	2.26(0.323)
	No	112(12.79)	74(8.45)	690(78.77)	
Knowledge of STI transmission	Good	63(11.52)	51(9.32)	433(79.16)	4.11(0.128)
	poor	83(15.51)	42(7.85)	410(76.64)	
Knowledge of STI symptoms	Good	66(10.95)	41(6.80)	496 (82.26)	14.96(0.001)^+^
	Poor	80(16.70)	52(10.86)	347(72.44)	
Knowledge of STI complication	Good	45(10.66)	29(6.87)	348(82.46)	8.34(0.015)^+^
	Poor	101(15.30)	64(9.70)	495(75.00)	
Knowledge of STI prevention	Good	86(12.74)	40(5.93)	549(81.33)	18.33(0.001)^+^
	Poor	60(14.74)	53(13.02)	294(72.24)	
Loss to follow up	Yes	86(13.29)	58(8.96)	503(77.74)	0.31(0.857)
	No	60(13.79)	35(8.05)	340(78.16)	
***Psychosocial factors***					
Perceived stigma to PN	High	55(14.10)	53(13.59)	282(72.31)	20.32(0.001)*
	Low	91(13.15)	40(5.78)	561(81.07)	
Intention to notify partner	Unwilling	92(25.14)	43(11.75)	231(63.11)	77.50(0.001)^+^
	Willing	54(7.54)	50(6.98 )	612(85.47)	
Self-efficacy to prevent reinfection	High	77(11.53)	49(7.34)	542(81.14)	10.56(0.005)^+^
	Low	69(16.67)	44(10.63)	301(72.71)	
P-value < 0.05					

**Table 2:** Results of the multiple POM using perceived risk of reinfection for STIs, North Ethiopia, 2015

Co-variable	Coefficient	SE	P- value	Odds Ratio	95% CI	Single score test (p value)
Intercept 1	-2.034228	0.35	-	-	-	-
Intercept 2	-1.367806	0.34	-	-	-	-
Current age (<24 years as reference)						
>25 years	0.42	0.17	0.01	1.52	(1.10-2.11)	0.862
Participant sex (Male as reference)						
Female	0.38	0.16	0.02	1.47	(1.06-2.05)	0.645
Marital status (Married as reference)						
Single	0.12	0.24	0.62	1.13	(0.70-1.83)	0.490
Educational status (Illiterate as reference)						
Primary	0.25	0.23	0.27	1.28	(0.82-1.99)	0.230 0.580
High school+	0.50	0.23	0.03	1.65	(1.05-2.60)	
Residence (Urban as reference)						
Rural	-0.06	0.18	0.73	0.94	(0.66-1.34)	0.002
Partnership type (Regular as reference)						
Casual	-0.07	0.24	0.78	0.93	(0.57-1.52)	0.203
Number of partners (Multiple as reference)						
Single	0.60	0.20	0.00	1.81	(1.21-2.73)	0.346
Know STI symptoms (Good as reference)						
Poor	0.41	0.16	0.01	1.51	(1.10-2.08)	0.880
Know STI prevention (good as reference)						
Poor	0.40	0.16	0.01	1.49	(1.10-2.04)	0.003
Know STI complication (good as reference)						
Poor	0.01	0.17	0.94	1.01	(0.72-1.42)	0.811
Perceived stigma to PN (High as reference)						
Low	0.26	0.16	0.08	1.30	(0.96-1.78)	0.002
Intention towards PN (Willing as reference)						
Unwilling	1.09	0.17	0.00	2.97	(2.11-4.20)	0.095
Self-efficacy to PR (High as reference)						
Low	-0.24	0.17	0.16	0.78	(0.56-1.09)	0.902
Score test for the proportional odds assumption: Chi-square =35.47, df = 13, p-value = 0.001 Goodness-of-fit test of overall model (Likelihood Ratio): Chi-square= 123.94, df = 13, p-value = 0.001, Pseudo R2= 0.085						

**Table 3:** Results of the PPOM using perceived risk of reinfection for STIs, North Ethiopia, 2015

Co-variable	Comparisons					
	Low Vs. Medium or High risk			Low or Medium Vs High risk		
	Odds Ratio	95% CI	P value	Odds Ratio	95% CI	P value
**Current age (Ref. ≤24 years)**						
≥25 years	1.52	1.09-2.12	0.012^+^	1.52	1.09-2.12	0.012^+^
**Sex of participant (Ref. male)**						
Female	1.49	1.07-2.08	0.017^+^	1.49	1.07-2.08	0.017^+^
**Marital status (Ref. married)**						
Single	1.13	0.69-1.84	0.612	1.13	0.69-1.84	0.612
**Education (Ref. Illiterate)**						
Primary	1.10	0.68-1.77	0.692	1.39	0.89-2.18	0.144
High school+	1.68	1.07-2.65	0.023^+^	1.68	1.07-2.65	0.023^+^
**Residence (Ref. Urban)**						
Rural	1.24	0.79-1.94	0.334	0.86	0.60-1.23	0.412
**Partnership type (Ref. Regular)**						
Casual	0.95	0.58-1.55	0.853	0.95	0.58-1.55	0.853
**No of partners (Ref. multiple)**						
One	1.82	1.20-2.74	0.004^+^	1.82	1.2-2.74	0.004^*^
**Knowledge of STI symptoms** **(Ref. Good)**						
poor	1.54	1.12-2.13	0.008^+^	1.54	1.12-2.13	0.008^+^
**Knowledge of STI prevention** **(Ref. Good)**						
poor	1.17	0.80-1.71	0.394	1.60	1.17-2.21	0.004^+^
**Knowledge of STI complication (Ref. Good)**						
poor	1.00	0.71-1.41	0.985	1.00	0.71-1.41	0.985
**Perceived stigma to notify partner (Ref. High)**						
Low	0.99	0.67-1.45	0.980	1.42	1.04-1.95	0.027^+^
**Intention to notify partner** **(Ref. unwilling)**						
Willing	3.01	2.13-4.25	0.000^+^	3.01	2.13-4.25	0.001^+^
**Self-efficacy to perceived risk (Ref. high)**						
Low	0.78	0.56-1.08	0.146	0.78	0.56-1.08	0.146
Score test for the proportional odds assumption: Chi-square = 13.05, df = 10, p-value = 0.2211 Goodness-of-fit test of overall model (Likelihood Ratio):Chi-square = 155.58, df = 18, p-value = 0.001, Pseudo R2 = 0.106 ^+^p-value < 0.05						

### Ethical consideration

This study was approved by the Research Ethics Review Committee of the College of Health Science, Mekelle University, Ethiopia. Patients were informed about the objective of the study and verbally consented prior to the interview. Patients were interviewed in private room by same gender interviewer after obtained the routine care. Participants were also informed the right to freely decline from the study at any time.

## Results

A total of 1082 STI patients were involved in the study. Of the respondents, 843(77.91%) had high perceived risk of STI reinfection. As shown in Table 1, all considered variables except condom use in the last three months, new partner in the last three months, and knowledge of STI had significant association with risk perception for STI reinfection. The assumption for POM was violated as indicated by the score test value of 0.001 (Table 2).

A multivariate analysis of pooled sample using the PPOM is indicated in Table 3. Willing to notify partner was found to be a strong predictor of risk perception to reinfection. Those index cases willing to notify partners had higher odds to perceive low risk compared to those unwilling to notify (AOR=3.01, 95% CI: 2.13-4.25). In addition, female index cases had higher odds to have low or medium perceived risk of reinfection for STIs compared to males (AOR=1.49, 95% CI: 1.07-2.08); similarly low or medium perceived risk of reinfection for STIs showed higher odds among those with secondary education and above compared to no education (AOR=1.68, 95% CI: 1.07-2.65), among those aged > 25 years compared to the younger ages (AOR=1.52, 95% CI:1.09-2.12), among those having single sexual partner compared to those with multiple partners (AOR=1.82, 95% CI: 1.20-2.74), among those having poor knowledge of STI symptoms compared to their counter parts (AOR=1.54, 95% CI: 1.12-2.13), among those with poor knowledge of STI prevention (AOR=1.60, 95% CI: 1.17-2.21) and among those with low perceived stigma to notify partner compared to their counter parties (AOR=1.42, 95% CI: 1.04-1.95).

## Discussion

This study examined risk perception of STIs reinfection and its relationship with socioeconomic, behavioral and psychosocial factors in the context of Ethiopia. Overall, the proportion of index cases who perceived risk to reinfection was found to be high (77.91%). The low perceived risk of reinfection was found higher among respondents willing to notify partners compared to those unwilling in this study. This relationship was not clearly suggested in previous studies. However, this can be possibly explained as being willing to notify partner may indicate the confidence and self efficacy of index cases on their partners risk status which inspire them to perceive less risk of reinfection. Besides, the intent to continue partnership among those willing to notify partner may also attribute low risk perception. In fact, perceived risk influences the willingness to engage in preventive behaviors such as notifying a partner [17]. However, a study showed that patients’ willing to inform relatives did not significantly increase with high risk perception [18, 19] indicating that high perceived risk itself may reduce intention to inform partner because of negative believes and consequences.

Gender has a significant role in perceived risk of reinfection which showed female respondents were more likely to have low perceived risk of reinfection compared to their counter parts. Our finding is aligned with a previous study on HIV risk perception [20], that reported most women perceived themselves to be at no or low risk for HIV infection. This might be related to the fact that females tend to trust their intimate partners which in turn reduces the risk perception [21]. However, inconsistency in finding was also reported in women who were more likelihood to perceive high STI/HIV risk compared to men in Sweden and USA [16, 22]. Possibly, being aware of the higher susceptibility to contract STIs/HIV could influence women to perceive more risk [23]. In this study, patients older than 25 years and above were more likely to have low perceived risk of reinfection compared to younger ages. Possibly, exposure to sexuality related risks may decrease with aging that again reduce individual’s risk perception [24]. In contrast, a previous study has shown that majority of young people perceive low risk [25] because of the fact that they don’t realize or acknowledge that they are at risk of reinfections.

A striking inverse relationship between perceived risk of reinfection and level of education was found in this study which is similar to a study conducted by Rowe J et al [26]. Education was linked to increased sense of control that leads to lower perceived risks [27]. Furthermore, educational attainment may not necessarily translate to knowledge of possible STI reinfection, which is vital for increased risk perception [24]. Importantly, educated individuals may practice safer behavior because of acquired knowledge of subsequent risks which may impact in low perceived risk. In contrast, a positive correlation between perceived risk and level of education was reported in previous [28, 29]. In light of this, highly educated individuals perceive more risk since they have better information and awareness about the subsequent possible risks.

Patients with low perceived risk were also more likely than those with high perceived risk to have one sexual partner in the current study. This is in agreement with previous studies [16, 25] that report an elevated risk perception was common among respondents with multiple partners. Though the perceived risk is higher in HIV than STIs, a study on HIV also revealed that having multiple sexual partners was found significant predictor of perceived risk of HIV reinfection [30]. This might cause participants to perceive themselves at risk because they have unstable sexual relationships or they were aware of their partner’s sexual practice [31]. It was also suggested that people with multiple sexual partners agreed up on their risk but behavioral actions to prevent risks depend on social and individual factors [32]. Index cases with poor knowledge of STI symptoms and prevention were also found to be correlated with low perceived risk of STI reinfection in this study. This is in agreement with the previous study [33]. A study on HIV also showed that individuals with inadequate knowledge of HIV reinfection were less likely to perceive risk of HIV reinfection [30]. This shows that poor knowledge is a barrier to prevent reinfection. This study also showed that perceived stigma to notify partners was significantly associated with perceived risk of reinfection. Those who perceived low stigma were more likely to perceive low risk of reinfection. Stigma has been reported as barrier of PN [34] and perceived risk of reinfection is also an indicator of partner notification. The positive association between low perceived stigma and low perceived risk of reinfection may indicate infections or disease with low stigma could lead individuals to perceived low risk [35]. This implies the need to aware STI patients about the risk of reinfection during daily practice of STI management.

### Strengths and limitations of the study

This study has some limitations. A single item question was used to measure perceived risk of reinfection for STIs. This may decrease the validity and internal consistency of our measure compared to scale construct. However, previous studies have used single-item variables rather than scales [15, 36] . In addition, since the study was conducted among patients seeking STI care in selected health facilities, the response of study participants might have favorably biased and overestimate risk perception. Assessment of patient’s willingness on partner notification was based on subjective responses from the study participants. The study had no objective way of validating the truth of information provided by respondents. This might result in over reporting of willingness to notify partner by index cases because of social desirability bias. However, interviews were administered by trained and same sex interviewers in face-to-face and one-on-one settings to ensure confidentiality to minimize bias. The study was also limited in that it relied on self-report, and is therefore subject to reporting bias. Despite these limitations, relatively large sample of STI patients were examined. A more robust and fitted model (PPOM) was used for analysis as an alternative approach when the POM was not satisfied.

## Conclusion

In conclusion, our study is among the few to examine the social, behavioral and psychosocial characteristics of patients that predict perceived risk of STI reinfection. We found willing to notify partners as a main determinant of low perceived risk. Other social and behavioral variables such as education, age and number of partners were also found significantly associated with perceived risk. These findings highlight the need for interventions that enhance partner notification among partners to reduce the exposure to risk of STI reinfection.

### What is known about this topic

- Reinfection after treatment for sexually transmitted infections indicates the existence of risk behavior after treatment and it is a serious problem (Low N, 2013);
- Variation in magnitude of reinfection for STIs by type of infection;
- Perceived risk as important measure to assess actual behavior (Taylor-Goby P, 2006).

### What this study adds

- *Magnitude of risk perception to reinfection* among patients treated for sexually transmitted infection;
- Social, behavioral and psychosocial *predictors of risk perception to reinfection* among patients treated for STIs.

## Competing interests

The authors declare no competing interest.
